# Pulmonary Langerhans Cell Histiocytosis and Diabetes Insipidus in a Young Smoker

**DOI:** 10.1155/2016/3740902

**Published:** 2016-04-11

**Authors:** K. Earlam, C. A. Souza, R. Glikstein, M. M. Gomes, S. Pakhalé

**Affiliations:** ^1^Division of Respirology, The Ottawa Hospital, University of Ottawa, Ottawa, ON, Canada K1H 8L6; ^2^Department of Medical Imaging, The Ottawa Hospital, University of Ottawa, Ottawa, ON, Canada K1H 8L6; ^3^Department of Pathology and Laboratory Medicine, The Ottawa Hospital, University of Ottawa, Ottawa, ON, Canada K1H 8L6

## Abstract

Langerhans cell histiocytosis is characterized by the abnormal nodular proliferation of histiocytes in various organ systems. Pulmonary involvement seen in young adults is nearly always seen in the context of past or current cigarette smoking. Although it tends to be a single-system disease, extrapulmonary manifestations involving the skin, bone, and hypothalamic-pituitary-axis are possible. High resolution CT (HRCT) of the thorax findings includes centrilobular nodules and cysts that are bizarre in shape, variable in size, and thin-walled. Often the diagnosis can be made based on the appropriate clinical presentation and typical imaging findings. Treatment includes smoking cessation and the potential use of glucocorticoids or cytotoxic agents depending on the severity of disease and multisystem involvement.

## 1. Case Presentation

A 19-year-old man was referred for further management given a history of recurrent pneumothoraces and a recent CT of the thorax which revealed multiple bilateral pulmonary cysts.

His past medical history included a surgically resected osteochondroma from the medial aspect of his right tibia a year ago, a remote appendectomy, and a childhood diagnosis of asthma. He did not take any medications regularly. He smoked half a pack of cigarettes a day for the last six years and also smoked seven marijuana joints per day. He denied consuming alcohol or any other illicit drugs. He worked in the family restaurant and both of his parents smoked tobacco. Other than chest pain and dyspnea experienced at the time he was diagnosed with pneumothoraces, he denied any symptoms. He did describe drinking about 15 liters of water a day due to a dry mouth. He denied any bone pain, skin rashes, or abdominal pain. Review of systems was unremarkable.

Prior to referral, he had presented to hospital with a spontaneous right-sided pneumothorax on two occasions and bilateral pneumothoraces on one occasion. The third time he was treated surgically and a video-assisted thoracoscopic bullectomy with apical pleurectomy of the right lung was performed. He was found to have asymptomatic recurrence of a right-sided pneumothorax when seen in follow-up and was treated successfully with chemical pleurodesis in the form of bleomycin.

On examination, he was afebrile, blood pressure was 115/75, heart rate was 88 beats/min, and respiratory rate was 16 breaths/min. Weight was 93.5 kg and height 183 cm, resulting in a BMI of 27.9. The cardiovascular examination was normal except for a loud P2. The respiratory examination revealed normal breaths sounds bilaterally. The remainder of the examination was unremarkable.

**Figure 1:**
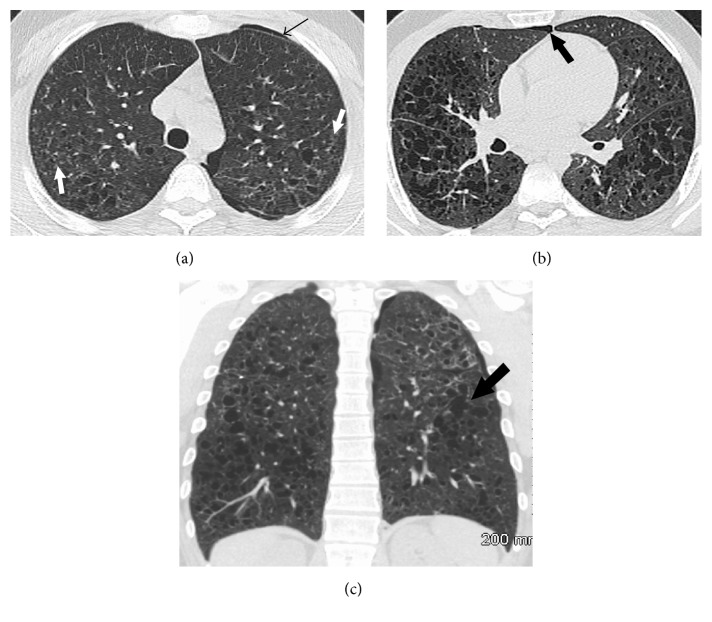
(a) Axial CT scan of the chest with lung window settings at the level of the upper trachea demonstrates multiple bilateral thin-walled cysts and a few ill-defined centrilobular nodules (white arrows). Note a small left pneumothorax (thin black arrow). (b) Axial CT scan of the chest with lung window settings demonstrates bilateral thin-walled cysts, some with bizarre shapes such as in the left lower lobe. Note a small residual right-sided pneumothorax (arrow). (c) Coronal CT reformats with lung window settings demonstrate bilateral lung cysts, some with bizarre shapes (arrow) involving mainly the mid and upper lung zones with relative sparing of the costophrenic angles.

Pulmonary function tests revealed airflow obstruction with an FEV_1_/FVC ratio of 43% (normal ≥ 70%), an FEV_1_ of 2.14 L (44% of predicted), and a FVC of 4.95 L (86% of predicted). There was no evidence of restriction and the diffusion capacity for carbon monoxide (DLCO) was decreased at 60%. His spirometry did demonstrate significant reversibility postbronchodilator. A CT of the thorax performed prior to referral showed bilateral thin-walled pulmonary cysts of variable sizes and irregular shapes (see the following list). There were a few ill-defined centrilobular nodules and no consolidation, pleural effusions, or lymph node enlargement (Figures 1(a), 1(b), and 1(c)).

The following are the differential diagnoses of cystic lung disease:

 Langerhans cell histiocytosis
 Lymphoid interstitial pneumonia
 Neurofibromatosis
 Birt-Hogg-Dubé syndrome
 Pneumatoceles (due to prior* Pneumocystis carinii* pneumonia)
 Tuberous sclerosis
 Lymphangioleiomyomatosis

**Figure 2:**
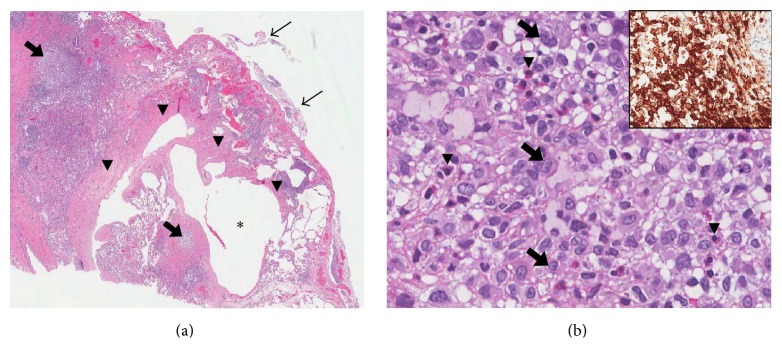
(a) The wedge lung biopsy shows cellular nodules (thick arrows) and an irregular fibrous scar (arrowheads) delimiting a cystically dilated airspace (*∗*). Note the flimsy adhesions on the pleural surface, secondary to the pneumothorax (right upper corner, thin arrows)—hematoxylin and eosin stain, 11x. (b) Higher magnification of a cellular nodule reveals numerous histiocytes with folded nuclei (arrows) in an eosinophilic inflammatory background (arrowheads depicting eosinophils)—hematoxylin and eosin stain, 400x. The inset shows strong expression of the immunohistochemical marker CD1a by the histiocytes (brown staining)—CD1a immunohistochemical stain, 110x.

**Figure 3:**
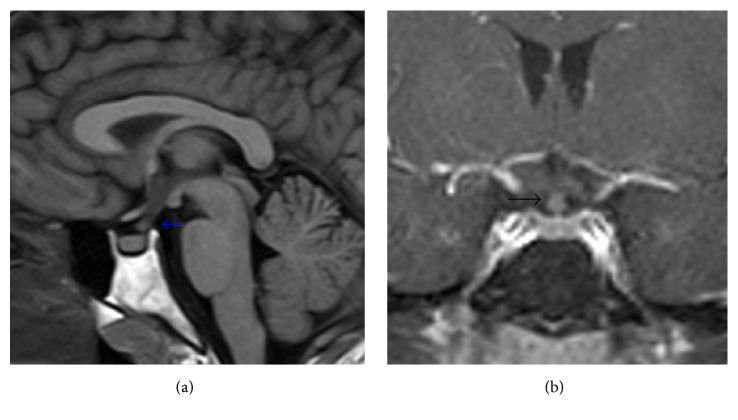
(a) Sagittal T1-weighted MRI of the sella turcica showing a thick pituitary stalk (arrow). Please note the absence of expected hyperintense (bright) signal of the neurohypophysis. (b) Coronal T1-weighted post-Gd MRI showing a thick pituitary stalk (arrow).

The patient's clinical presentation and investigations were consistent with the diagnosis of pulmonary Langerhans cell histiocytosis (PLCH) and possibly concurrent asthma. In fact, a previous bullectomy specimen showed histological features consistent with PLCH (Figures 2(a) and 2(b)). Given his history of polydipsia, he underwent a brain MRI which showed focal nodular thickening with enhancement of the infundibulum with otherwise normal size of the pituitary gland; findings were found to be consistent with posterior pituitary involvement (Figures 3(a) and 3(b)). Subsequent bloodwork showed a high-normal plasma sodium concentration of 142 mmol/L and a urine osmolality of 91 mmol/k, less than the plasma osmolality of 294 mmol/k. He did not have evidence of any other endocrinopathy on bloodwork. An echocardiogram did not show evidence of pulmonary hypertension. Given his history of a bone tumor resection, we obtained pathology results from the treating hospital which confirmed that the tumor was in fact an osteochondroma and not consistent with Langerhans cell histiocytosis.

He was treated with an inhaled corticosteroid and long-acting bronchodilator combination puffer. With desmopressin, his water intake reduced dramatically to 1-2 L/day. He presented again a year later with bilateral spontaneous pneumothoraces (left larger than right) and was treated with left-sided pleurodesis in the form of doxycycline. After a couple of relapses, he quit smoking after using varenicline and has remained smoke-free for 12 months at last follow-up. His latest FEV_1_ is 2.98 L (58% of predicted) with an FEV_1_/FVC ratio of 64%.

## 2. Discussion

Langerhans cell histiocytosis (LCH) refers to a group of disorders characterized by the abnormal proliferation of histiocytes and formation of granulomas in various organ systems. The Histiocyte Society has provided a classification scheme that divides histiocytic disorders into three groups: (1) dendritic cell histiocytosis, (2) macrophage cell histiocytosis, and (3) malignant histiocytosis [1, 2]. LCH is part of group one and is further categorized according to the extent of organ involvement at presentation. The acute disseminated multisystem form of LCH (previously known as Letterer-Siwe syndrome) usually manifests in children under the age of three years and in the majority of cases has a fatal course. Multifocal LCH (previously known as Hand-Schuller-Christian syndrome) is primarily seen in infants and children and has a more favourable course. Single-system disease usually presents in young adults and commonly involves the lungs, brain, or bone. Isolated pulmonary LCH (PLCH) in adults is nearly always seen in the context of past or current cigarette smoking [1, 2]. Pulmonary involvement can be seen in patients with multiorgan disease, but it tends not to be the main organ involved. However, when there is predominant lung involvement, it is considered a poor prognostic factor [3].

There is limited epidemiological data regarding PLCH, given that it is relatively rare. It typically affects young adults between the ages of 20 and 40 years, in a similar proportion amongst males and females, when considering the changes in gender smoking habits over time [4]. At least 90% of patients with PLCH are current smokers [5].

Typically, patients present with nonspecific symptoms such as nonproductive cough or dyspnea which are often attributed to smoking. In 10–15% of patients, spontaneous pneumothorax is the first manifestation of the disease. It may be bilateral and tends to recur, as illustrated in this case report [5]. Constitutional symptoms are seen less often and hemoptysis is unusual. Pulmonary function tests usually demonstrate a decreased diffusion capacity for carbon monoxide (DLCO), although mixed ventilatory defects are also common [5]. Approximately 25% of patients are asymptomatic at the time of presentation and abnormalities are incidentally noticed on chest radiograph.

Although PLCH is usually a single-system disease seen in a young adult smoker, it is important to consider extrapulmonary disease manifestations as well. Skin lesions, cystic bone lesions, and diabetes insipidus with polyuria and polydipsia are the commonest, as illustrated here. Liver and lymph node involvement are less common [4]. Hepatomegaly is suggestive of liver involvement and combined with other liver enzyme abnormalities should prompt further investigation with diagnostic imaging. The risk is that of LCH infiltrating the biliary ducts leading to cholestasis, which can progress to sclerosing cholangitis [6].

Abnormalities seen on chest radiograph vary with the stage of disease. The earliest change is a bilateral reticular or reticulonodular pattern involving the mid and upper lung zones. Cyst formation follows as the disease progresses. Lymph node enlargement and pleural effusions are rare. High resolution CT (HRCT) of the thorax is much more accurate to identify and characterize the lung parenchymal changes. In the vast majority of patients, HRCT demonstrates cystic lesions usually less than 10 mm in diameter with a predominant mid and upper lung distribution and relative sparing of the costophrenic angles [7]. Cysts may have thin or thick walls and although many may be round, they often have bizarre shapes, being bilobed, cloverleaf shaped, or branching in appearance. In the majority of cases, small nodules are also present and vary considerably in number, probably depending on the activity of the disease [8]. PLCH has a predictable radiological evolution, which mirrors the pathological changes (see below), and starts with centrilobular nodules that undergo cavitation with formation of thick-walled cysts and, ultimately, thin-walled cysts. Such HRCT findings in combination with a suggestive clinical presentation are sufficient to establish a confident presumptive diagnosis and surgical biopsy is usually reserved for atypical cases.

Biopsy of a lung lesion or any tissue involved by the disease is appropriate. The pathological hallmark of the initial (or cellular) phase of PLCH is the nodular accumulation of Langerhans cells in an eosinophilic inflammatory background which takes place in and around small airways [8]. Langerhans cells present folded nuclei and consistently express the immunohistochemical marker CD1a. This cellular proliferation is associated with tissue damage and secondary wound healing with the formation of irregular centrilobular scars, which is the hallmark of the late (or healing) phase. Therefore, the “cysts” seen in radiology are rather cystic-like changes and the “cavitations” represent remodeling of the fibrotic scar tissue. Lesions in different phases coexist and smoking-related changes, such as centrilobular emphysema and respiratory bronchiolitis, are frequently noted in the adjacent lung parenchyma. The cellular lesions tend to disappear after smoking cessation but the scars are permanent [9].

Given the low incidence of PLCH, data regarding treatment for this disease is largely derived from large case series, case reports, and expert opinion. Since the only epidemiological factor associated with PLCH has been cigarette smoking, smoking cessation is a critical part of management. This alone can result in resolution or improvement of the disease in some cases [5, 10]. Since most patients with PLCH will either be asymptomatic at presentation or remain in stable condition with no treatment, additional therapies have to be considered on a case-by-case basis. In the setting of symptomatic nodular lung disease, experts have suggested the use of glucocorticoids in hope that this treatment will resolve the inflammatory lesions and prevent them from progressing to cyst formation. Cytotoxic agents (i.e., vinblastine, methotrexate, and cyclophosphamide) are reserved for patients with severe multisystemic LCH [5, 10]. Certainly one should also remain vigilant for the possibility of pneumothorax in these patients. Lung transplantation is also an option in the setting of progressive disease or the development of pulmonary hypertension.

There is no long-term prospective data on the outcomes of patients with PLCH and the clinical course of the disease is unpredictable. Various factors have been associated with adverse outcomes, including multisystem involvement, prolonged constitutional symptoms, markedly reduced diffusing capacity, severe obstructive disease, old age, and severe pulmonary hypertension. Other malignancies, such as lymphoma and myeloproliferative neoplasms, have been noted to occur at a higher frequency in these patients as well [4, 5]. It is expert opinion that these patients be followed up in long term in order to detect exacerbation of respiratory disease and be monitored for complications. Pulmonary function tests and repeat HRCT of the thorax are potential ways to monitor progression.

## Additional Points

*Learning Objectives*

- To recognize the possible extrapulmonary manifestations of pulmonary Langerhans cell histiocytosis (PLCH).
- To understand and recognize the radiological manifestations of pulmonary Langerhans cell histiocytosis (PLCH).

CanMEDS competency is Medical Expert.

*Pretest*

1. What are the most common extrapulmonary manifestations of PLCH?
2. What are the computerized tomography (CT) manifestations of PLCH?
3. What is the pathological hallmark of PLCH?

*Posttest*

1. What are the most common extrapulmonary manifestations of PLCH?
   The most common extrapulmonary manifestations are skin lesions, cystic bone lesions, and diabetes insipidus from posterior pituitary involvement.
2. What are the computerized tomography (CT) manifestations of PLCH?
   The predominant findings include cysts of variable sizes and shapes with a predominant mid and upper lobe distribution. Small centrilobular nodules are often present and have similar distribution.
3. What is the pathological hallmark of PLCH?
   The presence of centrilobular nodules of Langerhans cells in an eosinophilic background and irregular fibrous scars. The Langerhans cells have characteristic morphological features and express the immunohistochemical marker CD1a.
